# Bottom-Up and Top-Down Effects Influence Bruchid Beetle Individual Performance but Not Population Densities in the Field

**DOI:** 10.1371/journal.pone.0055317

**Published:** 2013-01-29

**Authors:** Isabelle Zaugg, Betty Benrey, Sven Bacher

**Affiliations:** 1 Unit of Ecology & Evolution, Department of Biology, University of Fribourg, Fribourg, Switzerland; 2 Institut de Biologie, University of Neuchâtel, Neuchâtel, Switzerland; Natural Resources Canada, Canada

## Abstract

Plant quality (bottom-up) and natural enemies (top-down) can influence the individual performance of herbivorous insects on their host plants, but few studies measured at the same time the influence on population densities in the field. We investigated if plant quality of different wild common bean populations, *Phaseolus vulgaris* L. (Fabaceae), affects the performance of the bean weevil, *Acanthoscelides obtectus* (Say) (Coleoptera: Bruchidae), and one of its enemies, the ectoparasitoid *Dinarmus basalis* (Rondani) (Hymenoptera: Pteromalidae), in controlled laboratory experiments. Additionally, we examined if parasitoids influence the beetles' development and if increased individual beetle and parasitoid fitness lead to higher field population densities. We show that bean quality and parasitoids affected individual bean weevil performance under laboratory and field conditions. In the presence of parasitoids, fewer and smaller beetles emerged. However, beetle and parasitoid performance were not correlated. Increased individual performance was not leading to higher population densities; we found no correlations between measured performance components and beetle field infestation levels or parasitism rates. We conclude that bottom-up or top-down effects measured at the individual level do not always translate into population effects; therefore it is important to discriminate between effects acting on individual insects and those acting on populations.

## Introduction

The importance of plant-mediated effects (bottom-up) and natural enemies (top-down) in determining insect herbivore abundance on plants has been and is still controversially discussed [1]–[5]. Recently it has become more evident that those factors do not act individually in shaping insect communities, but are integrated with other factors like the herbivores' life-history strategies, location and time [2], [3], [6]. Plant secondary metabolites can affect herbivore performance via e.g. survival and growth rates, allocation of resources to eggs and egg quality, which can lead to bottom-up regulation of herbivores [7]–[10]. Differences in food plant quality can be found among different plant genotypes or even among plant individuals, thus herbivores are expected to choose high quality hosts in order to maximise their fitness [11]–[14].

The quality of host plants as food does not only affect herbivores, but can also influence their natural enemies either directly (if enemies are directly exposed to chemical compounds) or indirectly, e.g. via altered host size [15]–[17]. Changes in host plant quality can thus cascade up to higher trophic levels and influence performance and abundance of parasitoids or even hyperparasitoids of herbivores [18]–[20]. Hunter [7] has reviewed indirect effects of host plants on parasitoids through altered host size, herbivore growth rate (apparency) and herbivore chemistry or vigour. He found that many studies show that preference or performance of parasitoids is linked with the size of herbivores, but few consider the role of plant quality in generating the variation in herbivore traits. More and larger herbivore hosts on high quality plants can produce more parasitoids of larger size with greater longevity and egg loads [21], which should lead to higher parasitism rates. Therefore plant nutritional quality could also affect population densities by increasing individual fitness of herbivores and their natural enemies, leading to larger parasitoid populations or higher parasitism rates on high quality host plants [9], [20], [22], [23].

Some studies have investigated the influence of variation in plant nutritional quality on the individual performance of herbivores or natural enemies in the laboratory, but few considered simultaneously the impact of plant quality on populations of herbivores and parasitoids in the field [24], [25]. Plant genotype has been shown to influence population dynamics or abundances of herbivores; whereas the influence on natural enemies is less pronounced. Kos et al. [20] have found an effect of chemistry and morphology in cultivated *Brassica* plants on the abundance of herbivores and natural enemies. In contrast, Newton et al. [9] reported no direct effect of plant genotype on numbers of natural enemies. Since evidence of host plant effects on field abundances of herbivores and natural enemies remain scarce, we wanted to assess those effects in common beans, *Phaseolus vulgaris* L. (Fabaceae).

Here, we first investigated in controlled laboratory experiments the effect of host plant nutritional quality on the performance of a herbivore and its parasitoid. In a second step, we examined if the benefit of nutritionally superior plants for the individual insects is also transferred to the population levels, i.e. if individual performance is correlated with field population densities of herbivores and parasitoids. We studied these multi-trophic effects in wild common beans, *Phaseolus vulgaris* L. (Fabaceae) and their main herbivore, bean weevils from the genus *Acanthoscelides* (Coleoptera: Bruchidae) and their parasitoids.

Apart from the main storage protein phaseolin, common bean seeds contain a family of closely related seed proteins (lectins and lectin-related proteins), who are considered to play a role in the plant's defence against attacks from herbivores [26]. In the intestinal tracts of herbivores, these defence proteins can disrupt cell walls and inhibit nutrient absorption [26]. It has been shown that seeds from wild *P. vulgaris* populations differ in their defence protein composition [27]–[29]. One of these defence proteins, arcelin, causes sub lethal effects in *A. obtectus* larvae, prolonging developmental time of beetles and reducing their weight [27], [28]. Velten et al. [30] have shown that arcelin does not have any direct effects on the development of the parasitoid *Dinarmus basalis* since parasitoids feed on the hemolymph of beetle larvae and therefore do not come into direct contact with arcelin. However indirect effects of the protein, like reduced quality of their larval hosts can influence the parasitoid performance [30].

In addition to direct lethal effects, predators or parasitoids can have indirect non lethal effects, which are expressed as changes of the prey's behaviour in order to avoid being killed (Lima [31]), for example a reduction of the feeding activity [32]–[36]. Such non-consumptive predator effects have been shown to influence herbivore population dynamics, and their effects on structuring predator-prey interactions may be as strong as or even stronger than the effects of direct consumption [31], [36]–[38]. In laboratory experiments, we investigated if the performance of *A. obtectus* is directly and indirectly affected by the presence of natural enemies by estimating the effect of *Dinarmus basalis* (Rondani) (Hymenoptera: Pteromalidae) on beetle weights and developmental times.

We examined several hypotheses:

1. Bean populations vary in their nutritional quality for bean weevils and therefore they should influence the performance of individual beetles.
2. We further expect that increases in individual beetle performance on good quality hosts translate into increased performance at the population level, i.e. higher field infestation rates.
3. Additionally we predict that not only lethal, but also non-lethal effects of the presence of parasitoids influence the performance/fitness of their hosts (trait-mediated predator effects). This should result in smaller beetles and longer developmental times when parasitoids are present.
4. Since the development of parasitoids should depend on the quality of their hosts, we expect wasps to grow larger on larger hosts. We thus predict to find correlations between sizes and developmental times of *A. obtectus* and their parasitoids.
5. Finally, we tested if parasitism rates (as an indicator for population densities) are higher on larger hosts and/or high quality bean populations in the field.

## Materials and Methods

### Bean collection and insect determination

Wild common bean, *P. vulgaris*, seeds were collected at different locations in Mexico, in the States of Colima, Jalisco, México, Michoacán, Morelos and Puebla from December till March 2009/2010. Bean populations were found in ruderal habitats, close to cultivated crops, along highways or in rocky areas at elevations from 1319 m.a.s.l. (meters above sea level) to 2159 m.a.s.l. 50–100 bean pods were collected from each of totally 26 populations (Table 1). Pods were shelled and seeds from each population were kept in separate containers at 27°C and 70% R.H. until bruchids and/or parasitoids emerged. Every second to third day, containers were checked for emerging insects.

**Table 1:** Field data from 26 sampled bean populations in Mexico.

bean population (State)	altitude (m.a.s.l.)^a^	latitude (dd mm ss.s)^b^	longitude (dd mm ss.s)^b^	infestation level Acanthoscelides spp. (%)	parasitism rates (%)	tibia length of female parasitoids [mm]	weight Acanthoscelides spp. [mg]
ARRVP (Michoacán)	1848	N19 12 29.7	W101 43 11.7	10.1	3.9	NA (N = 0)	2.52±0.59 (N = 30)
ATL (Puebla)	1762	N18 52 40.3	W98 24 17.4	5.7	8.9	0.611±NA (N = 1)	2.99±0.51 (N = 27)
AXO (Puebla)	1896	N18 54 54.7	W98 27 29.2	24.1	4.1	0.589±0.004 (N = 2)	2.99±0.72 (N = 30)
COP1 (Michoacán)	2091	N19 26 59.6	W101 46 41.6	0.5	0	NA (N = 0)	3.21±0.59 (N = 8)
COPSPVC1 (Michoacán)	2092	N19 26 73.7	W101 46 28.2	6.2	0	NA (N = 0)	2.87±0.81 (N = 24)
COY (Michoacán)	1704	N19 20 93.9	W100 24 38.2	9.4	29.1	NA (N = 0)	2.55±0.85 (N = 22)
CVC2 (Morelos)	1843	N18 58 41.9	W99 12 57.5	11.8	24.4	0.624±0.12 (N = 17)	2.45±0.70 (N = 30)
CVC4 (Morelos)	1871	N18 58 47.6	W99 12 46.8	3.0	13.0	0.579±0.13 (N = 2)	1.22±0.59 (N = 23)
CVC6 (Morelos)	1886	N18 58 53.8	W99 12 34.8	9.8	13.0	0.612±0.10 (N = 13)	1.72±0.70 (N = 30)
DMSP (Michoacán)	1476	N19 06 65.9	W101 44 36.5	1.3	55.9	0.482±NA (N = 1)	1.25±0.35 (N = 12)
FENCE (México)	1527	N18 54 54.1	W99 29 23.3	13.5	28.3	0.569±0.04 (N = 2)	1.78±0.51(N = 30)
HUYS1 (Morelos)	2039	N18 52 24.0	W98 42 49.2	6.4	2.9	NA (N = 0)	2.34±0.41 (N = 30)
ISA (Jalisco)	1541	N20 24 58.7	W102 25 06.1	0	0	NA (N = 0)	0
JBSS (Puebla)	2159	N18 52 35.8	W98 35 59.6	2.9	27.7	0.581±0.07 (N = 9)	1.62±0.34 (N = 9)
MALS3 (México)	1828	N18 57 07.7	W99 30 16.5	59.6	12.0	0.582±0.07 (N = 11)	1.73±0.55 (N = 28)
MSLII (Michoacán)	1716	N19 12 00.5	W101 44 30.8	31.7	30.9	NA (N = 0)	1.78±0.56 (N = 28)
MSLIV (Michoacán)	1716	N19 12 00.5	W101 44 30.8	4.7	21.1	0.656±NA (N = 1)	1.51±0.29 (N = 17)
PIL2 (México)	1682	N18 56 54.4	W100 08 40.2	6.6	39.5	NA (N = 0)	1.15±0.35 (N = 11)
POC2 (Michoacán)	2026	N19 30 37.2	W100 22 675	51.8	17.5	0.724±0.09 (N = 2)	1.67±0.32 (N = 30)
QUES (Colima)	1319	N20 54 05.9	W103 24 80.3	0	0	NA (N = 0)	0
SCH (Jalisco)	2023	N19 58 55.6	W103 00 94.2	6.8	21.2	0.769±NA (N = 1)	1.58±0.34 (N = 19)
SJSA (Morelos)	1830	N18 58 49.6	W99 00 35.5	11.9	0	NA (N = 0)	NA
SJSA5 (Morelos)	1862	N18 58 49.6	W99 00 27.3	34.7	4.7	0.554±0.1 (N = 3)	1.40±0.54 (N = 30)
TEM (México)	1936	N19 01 65.3	W100 02 47.4	17.4	13.0	0.677±NA (N = 1)	1.28±0.35 (N = 18)
TZNIP1 (Michoacán)	2071	N19 38 36.7	W101 32 74.7	46.7	20.8	0.561±0.05 (N = 2)	1.97±0.47 (N = 24)
VUL (Morelos)	1504	N18 57 59.6	W99 03 88.1	1.9	33.9	0.497±NA (N = 1)	2.06±0.94 (N = 20)

a m.a.s.l.: meters above sea level.

b GPS coordinates (dd: degrees; mm: minutes; ss: seconds).

“NA” indicates values which could not be computed.

For each bean population, the State of origin, GPS coordinates (latitude and longitude) and the altitude were recorded. Additionally field infestation levels of *Acanthoscelides* spp., the most dominant bruchid genus, were computed for each population by dividing the number of emerged beetles by the number of collected seeds. Parasitism rates were calculated for each bean population by dividing the number of emerged parasitoids by the number of potential hosts (sum of bruchids + sum of parasitoids). The mean tibia length ± SD of *H. missouriensis* females, the most dominant species emerging from field collected seeds, and the mean weight ± SD of *Acanthoscelides* spp. were measured for each bean population.

We determined the sex of the emerged beetles and differentiated between the genera *Zabrotes* and *Acanthoscelides*. Two *Acanthoscelides* species are commonly found on wild *P. vulgaris* in Mexico, namely *A. obtectus* (Say) and *A. obvelatus* (Bridwell) [39]. Those sister species are closely related, co-occur on the same host plants and are difficult to distinguish from each other by their external morphology; thus they have been confused for a long time [40], [41]. Apart from differences in voltinism (*A. obtectus* is multivoltine, *A. obvelatus* is univoltine), *A. obtectus* has found to be more frequent at lower altitudes (<1800 m.a.s.l.); whereas *A. obvelatus* prefers higher altitudes (>1800 m.a.s.l). However, the range of both species overlaps [41]. Due to the species' high ecological similarity and our sampling sites being at altitudes suitable for both (Table 1), we did not differentiate between the two *Acanthoscelides* species emerging from field collected seeds. Additionally we did not differentiate between *Zabrotes subfasciatus* (Boheman) and its close relative *Z. sylvestris* [42]. Parasitoids of the genus *Horismenus* were determined to species level with the help of a determination key and previously collected and determined specimen [43]. All other parasitoids emerged were determined to family level.

To measure variation in beetle sizes among different bean populations, we averaged for each population the weights of about each 8–15 randomly selected male and female *Acanthoscelides* spp., the main bruchid genus found in the field. Tibia lengths of female parasitoids of *Horismenus missouriensis*, the most dominant parasitoid species, were measured as a surrogate for their size [30]. Field infestation levels of *Acanthoscelides* spp. were calculated for each bean population by dividing the number of insects emerged by the number of seeds collected (wild bean seeds are very small compared to commercial beans, and usually are used by only one beetle; pers. obs.). Field parasitism rates were estimated by dividing the sum of parasitoids emerged by the sum of potential hosts, which was calculated as sum of emerged parasitoids + sum of emerged bruchids. Gregariousness and hyperparasitism were not considered.

### Performance experiment with Acanthoscelides obtectus and Dinarmus basalis

Seeds of all 26 wild bean populations and two commercial Mexican bean cultivars (“Pinto”, “San Lanzano”) were used for performance experiments with *A. obtectus* and the ectoparasitoid *Dinarmus basalis*, a solitary idiobiont ectoparasitoid on larvae and pupae of several species of grain and bean weevils [44]. Females of *D. basalis* are synovigenic, i.e. females mature eggs throughout adult life. Prior to the experiments, beans were stored at −20°C for two days in order to kill all potential insects inside seeds [45]. Beetles originated from beans collected at local Mexican markets and they have been reared in climate chambers on red kidney beans for several generations prior to the experiments. Parasitoids were provided by Christoph Lüthi of the Research Station Agroscope Reckenholz-Tänikon, Switzerland.

Performance experiments were carried out according to a modified protocol of Velten et al. [30]. *A. obtectus* eggs were obtained by carefully sieving beans on which newly emerged adult beetles have been depositing eggs for 1 day. *A. obtectus* females do not attach their eggs to the surface of beans, but scatter them singly or in clusters among seeds [46]. Hatching larvae can move freely among beans and choose their hosts. Small plastic containers (height: 4 cm, diameter: 2.5 cm) were filled with 5 g of undamaged seeds of similar size. 50 eggs were added to each container using a moistened brush. We conducted 8 replicates for each of the bean populations tested. After 21 days at 27°C and 70% R.H., when bruchid larvae had reached the third to fourth instar, two males and two females of 2–6 day old *D. basalis* were introduced to half of the containers (4 replicates per bean population). Prior to the experiments, freshly emerged parasitoids were kept in small plastic containers without hosts and provided with a drop of honey to enhance oogenesis [47]. Parasitoids were left on experimental seeds throughout their lifespan, assuring sufficient time for host handling and mating [48]. Containers with beetles were checked daily for newly emerged insects. Emerged insects were kept singly in Eppendorf tubes, killed by deep-freezing at −80°C and beetles were immediately weighed. Bruchid sex was determined by dissecting the genitalia [39].

Parameters recorded for each beetle individual were its weight as an indicator for its size, sex and its developmental time, measured as number of days until adult emergence. Beetle survival was calculated as the percentage of *A. obtectus* emergence for each container by dividing the number of emerged bruchids by the number of eggs. For each parasitoid we recorded the developmental time and determined its sex. As a surrogate for parasitoid progeny size, we measured the length of the left hind tibia of each female parasitoid [30].

### Data analysis

#### Performance experiments with *A. obtectus* and *D. basalis*

Statistical analyses on the performance of beetles were carried out in R, version 2.14.0 [49]. For analyses with the dependent variables “size” and “developmental time”, the individual beetles were the experimental units. For the analysis on “survival” (coded as binomial variable: survivors over dead, separate for males and females), the individual containers were the experimental units. Since data on individual beetles that emerged from the same container cannot be regarded as independent, we included the container as random variable in the analyses. The explanatory variables of all models were the bruchids' sex, if parasitoids were added or not and the bean population.

Data on *A. obtectus* weight showed no departure from the assumptions of normality and heteroscedasticity; thus they were modelled using a linear mixed effects model (function lme from the package nlme; [50]). An analysis of variance table (function anova) was used to investigate the effects of the fixed factors on beetles' weight. Data on developmental time were assumed to follow a Poisson distribution (first emergence counted as day zero) and were analysed with a generalized linear mixed effects model with a logarithmic link function (function lmer from the package lme4, version 0.999375–42; [51]). Overdispersion was accounted for by including beetle individuals as random variable in the model. An analysis of deviance table with type II error (function Anova from the package car, version 2.0–11; [52]), which uses a Wald Chi-square test, was used to investigate the effect of the explanatory variables on beetles' developmental time.

A generalized linear model (function glm), assuming a binomial distribution of the response variable and a logit link function, was used to analyse the number of successfully emerged beetles over the failures per container. Results were displayed as analysis of deviance table with type II error (function Anova from the package car).

Since parasitoids are depending in their development on the bruchid hosts, we investigated if the performance of bruchid beetle individuals from the experiments showed an association with parasitoid performance using Spearman rank correlation tests (function cor.test). For this test, we computed for each bean population the mean bruchid weight (separately for males and females) and the mean bruchid and parasitoid developmental times from the containers to which parasitoids were added. The bruchid weight was tested for correlation with the size of parasitoid females and the mean beetle and parasitoid developmental times were tested for correlations.

#### Correlations between performance of insects in the laboratory experiments and the field

To test for an association between performance of bruchids on the same bean populations in the laboratory experiments and in the field, we used Spearman rank correlation tests. For each bean population we calculated the mean weight of *A. obtectus* males and females, the mean developmental time and the mean survival in the laboratory experiments. As surrogate for beetle performance in the field, we used for each bean population the mean weight of emerged *Acanthoscelides* spp. males and females. For parasitoids, we tested for correlations between mean female tibia sizes of *D. basalis* in the laboratory experiment and those of *H. missouriensis*, the most dominant parasitoid species in the field, on the same bean populations.

#### Correlations between host and parasitoid performance in the field

We hypothesized that parasitoids in the field should depend in their development on the quality of their beetle hosts; therefore we tested for a correlation between the mean size of *H. missouriensis* and the mean size of *Acanthoscelides* spp. males and females per bean population. Additionally we used Spearman correlations to investigate if parasitism rates were higher in bean populations with higher bruchid densities. To test if performance of *Acanthoscelides spp*. in the field is affected by parasitoid densities, we correlated the mean weight of beetle males and females per bean population with parasitism rates. Infestation rates (bruchids per bean, wasps per bruchid) are interpreted as indicators of population densities in the field (numbers per host, not per area).

#### Correlations between insect performance and population densities

Bean populations, which are of higher nutritional quality for bruchids and thus produce fitter individuals, could also allow larger insect population densities in the field. Therefore we tested for associations between performance of individual bruchids (laboratory and field) and their respective field infestation levels. We also investigated if there are correlations between parasitoid size in the field and parasitism rates. Correlations were carried out with Spearman's rank correlation test. We excluded populations where no insects emerged from the field-collected beans.

## Results

### Bean collection and insect determination

From 32′100 field-collected *P. vulgaris* seeds overall 10′884 insects emerged. We found insects emerging from seeds of all bean populations, except from populations “ISA” and “QUES”. 80.8% of the insects were bruchid beetles (Coleoptera: Bruchidae), with *Acanthoscelides* (98%) being the most abundant genus, followed by *Zabrotes* (2%). 12.6% of the emerged insects were hymenopterous parasitoids, with *Horismenus missouriensis* Ashmead (Hymenoptera: Eulophidae) being the most abundant parasitoid species found (40% of all parasitoids), followed by parasitoids of the genus *Lyrcus* (Hymenoptera: Pteromalidae) (36%) and *Horismenus depressus* Gahan (15%). Other hymenopteran parasitoids belonged to the families of Braconidae (8%), Eupelmidae (0.5%), Pteromalidae (0.3%), Eurytomidae (0.1%) and Torymidae (0.1%). The other 6.6% insects emerged from the beans belonged to the family of Apionidae (Coleoptera).

*Acanthoscelides* spp. females emerging from field-collected beans had an average weight of 2.1 mg±0.7 mg SD, males of 1.7mg±0.6 mg SD. Field infestation levels of *Acanthoscelides* spp. varied greatly between bean populations, with “MALS3” (59.6%) showing the highest and “COP1” the lowest levels (0.5%; Table 1). Likewise, parasitism rates in the field varied among bean populations with some populations having no parasitoids and “DMSP” having the highest rate (55.9%; Table 1).

### Performance experiment with Acanthoscelides obtectus and Dinarmus basalis

*A. obtectus* were able to develop on all bean populations tested with totally 5′847 individuals emerging from 13′600 eggs. Overall survival rate from egg to adult was 43% (overall sex ratio: males: 50.0%; females: 50.0%) (Table 2). 61.1% of all adult beetles emerged were from containers without parasitoids; whereas from containers with parasitoids 38.9% of adults emerged (p<0.001; Table 3). Adult bruchid emergence was not significantly different between sexes (Table 3), but we found significant variation among survival rates from different bean populations (p<0.001; Table 3). Apart from the bean cultivar “Pinto” (73.5%), the wild population “AXO” showed the highest parasitism rate (72.6%), whereas population “DMSP” showed the lowest (10%; Table 2).

**Table 2:** Overview of the laboratory performance of *Acanthoscelides obtectus* on seeds of 26 sampled bean populations from Mexico (N = 5′847).

bean population	*A. obtectus* (N)	bruchid weight [mg] ± SE	developmental time ± SE	survival rate ± SD	tibia length of female parasitoids [mm] ± SE	parasitoid females (N)	parasitism rates (%)
ARRVP	192	3.8±0.15	31.8±2.06	48.0±0.17	0.734±0.05	8	21.2
ATL	347	4.0±0.11	31.9±1.96	43.0±0.20	0.682±0.08	61	60.3
AXO	165	3.7±0.16	33.0±1.79	41.3±0.21	0.699±0.04	37	72.6
COP1	602	4.4±0.09	32.3±2.25	50.0±0.24	0.703±0.05	83	59.1
COPSPVC1	237	4.4±0.15	33.4±2.08	59.3±0.29	0.725±0.04	26	53.2
COY	150	3.4±0.16	31.7±2.26	37.3±0.14	0.710±0.07	15	39.2
CVC2	136	3.3±0.16	33.7±2.33	34.0±0.03	0.719±0.05	6	36.6
CVC4	121	3.1±0.17	33.8±2.57	30.3±0.08	NA	0	16.7
CVC6	189	3.9±0.15	33.7±2.32	47.3±0.14	0.698±NA	1	10.6
DMSP	110	2.8±0.17	33.4±2.32	27.5±0.06	NA	0	10.0
FENCE	189	3.8±0.15	32.1±1.80	47.3±0.17	0.644±0.10	17	48.4
HUYS1	152	3.6±0.16	32.8±2.74	38.0±0.10	0.668±0.07	11	37.8
ISA	159	3.8±0.16	39.4±2.50	45.4±0.17	0.561±0.13	4	35.6
JBSS	358	3.6±0.11	32.2±1.75	44.8±0.18	0.673±0.07	30	44.1
MALS3	165	3.6±0.16	32.4±2.32	41.3±0.14	0.671±0.13	5	22.2
MSLII	189	4.1±0.15	33.5±2.21	47.3±0.15	0.704±0.05	32	57.5
MSLIV	135	3.4±0.16	32.8±2.54	33.8±0.12	0.690±0.05	8	33.3
PIL2	198	3.6±0.15	33.6±2.28	49.5±0.13	0.741±0.05	13	29.3
POC2	182	4.0±0.15	31.6±2.32	45.5±0.16	0.701±0.04	7	38.2
QUES	70	2.9±0.20	37.9±3.28	17.5±0.09	0.683±0.05	8	57.1
SCH	359	4.0±0.11	34.0±2.34	44.9±0.16	0.693±0.06	48	47.4
SJSA	196	4.0±0.15	32.3±2.64	49.0±0.15	0.720±0.05	57	23.4
SJSA5	128	3.2±0.17	33.6±2.23	32.0±0.20	0.650±0.09	12	53.8
TEM	126	3.4±0.17	32.4±3.04	31.5±0.13	0.695±0.07	12	42.0
TZNIP1	436	4.8±0.11	32.9±2.04	54.5±0.23	0.715±0.06	61	49.0
VUL	106	3.4±0.17	33.7±2.25	26.5±0.11	0.724±0.05	2	26.7

N =  number of emerged *A. obtectus* or *D. basalis* females for each bean population

“NA” indicates values which could not be computed.

For each bean population the mean weight of emerged *A. obtectus*, the mean developmental time and the standard errors were recorded. For the survival rate of *A. obtectus*, the standard deviation was computed. In the case of *Dinarmus basalis,* the mean tibia length and the standard error were measured (N = 564). Parasitism rates were computed by dividing the number of emerged parasitoids by the number of potential hosts (sum of bruchids + sum of parasitoids).

**Table 3:** Performance experiments with *A. obtectus* on beans of 26 wild bean populations from Mexico.

performance experiments *A. obtectus*									
dependent variables:	weight			developmental time			survival rate		
explanatory variables:	DF^a^	F-value	p-value	Chisq^b^	DF^a^	p-value	Chisq^b^	DF^a^	p-value
sex	1	252.2	< 0.001***	138.4	1	<0.001***	n.s.	n.s.	n.s.
parasitoids added	1	16.3	< 0.001 ***	7.9	1	0.005**	99.8	1	<0.001***
bean population	27	21.5	< 0.001 ***	1129.9	27	<0.001***	127.2	31	<0.001***

a degrees of freedom.

b Wald Chi squared test.

Asterisks indicate significant values.

n.s. indicate not significant values.

50 eggs were added to each container (N = 8) and for each beetle its sex, weight and developmental time was determined. To half of the containers we added 2 pairs of *Dinarmus basalis* parasitoids. *A. obtectus* weight was analysed using an Anova table of the linear mixed effects model (lme) with bruchid weight as dependent variable and the container as random variable. Developmental time was analysed using an Analysis of deviance table (type II test) of the generalized linear mixed model (GLMM) with developmental time as dependent variable and container as random factor. Survival rate of *A. obtectus* was analysed using an Analysis of deviance table (type II test) of the generalized linear model (GLM). The dependent variable in the model was the number of successful emergences of beetles over the failures and the container was the random variable.

*A. obtectus* females were significantly larger than males (females: 3.8 mg±0.15 mg SE, males: 3.2 mg±0.15 mg SE; p<0.001; Table 3). Bruchids from containers without parasitoids were on average 0.14mg±0.05mg SE heavier than beetles from containers with parasitoids (p<0.001; Table 3). There also was significant variation among the weight of bruchids from different bean populations (p<0.001; Table 3).

Bruchid females needed on average 33.5±0.05 SE days to develop to adults; whereas males needed on average 32.5±0.05 SE days (p<0.001; Table 3). When parasitoids were added to containers, beetles emerged earlier (p = 0.005; Table 3; estimate: −0.02±0.01 SE). Developmental time of bruchids was varying among different bean populations (p<0.001; Table 3).

We found no significant correlation between performance of bruchid hosts and parasitoids in the experiment. The mean tibia length of parasitoid females in the experiment was not significantly correlated to the mean weight of bruchid hosts (Spearman's rank test: p = 0.26, rho = 0.255). We also found no significant association between *D. basalis* and *A. obtectus* developmental times (Spearman's rank test: p = 0.41, rho = −0.18).

### Correlations between performance of insects in the laboratory experiments and in the field

In both bruchid sexes, we found significant positive correlations between mean field weights and weights from the performance experiment (Table 4). We found no correlation between parasitoid sizes measured in the experiment (*D. basalis*) and in the field (*H. missouriensis*) (Spearman's rank test: p = 0.62, rho = 0.143).

**Table 4:** Correlation between laboratory and field performance of *Acanthoscelides* beetles.

	performance in the field:		
performance in the experiment:	weight males	weight females	infestation level
weight males	p = 0.01*; rho = 0.517	-	p = 0.29; rho = 0.227
weight males with parasitoids	p = 0.003**; rho = 0.59	-	p = 0.41; rho = 0.179
weight males without parasitoids	p = 0.04*; rho = 0.431	-	p = 0.21; rho = 0.270
weight females	-	p = 0.01*; rho = 0.513	p = 0.33; rho = 0.211
weight females with parasitoids	-	p = 0.13; rho = 0.328	p = 0.36; rho = 0.200
weight females without parasitoids	-	p = 0.001**; rho = 0.618	p = 0.90; rho = 0.027
developmental time	-	-	p = 0.41; rho = −0.182
developmental time with parasitoids	-	-	p = 0.56; rho = −0.127
developmental time without parasitoids	-	-	p = 0.34; rho = −0.208
survival rate	-	-	p = 0.43; rho = 0.172
survival rate with parasitoids	-	-	p = 0.55; rho = 0.132
survival rate without parasitoids	-	-	p = 0.64; rho = 0.104

Asterisks indicate significant values.

“-“parameters were not tested.

Spearman rank tests were performed to investigate whether parameters of performance experiments with *A. obtectus* correlate with field weights of *Acanthoscelides* spp. males and females and field infestation levels. Data for bean population “ISA” and “QUES” were not included in the analysis since no insects emerged from those seeds. P-values and the Spearman's rank correlation coefficient “rho” are indicated.

### Correlation of host and parasitoid performance in the field

In the field, the size of *H. missouriensis* parasitoids was not determined by the size of their hosts (Spearman's rank test: bruchid females: p = 0.72, rho = 0.097; males: p = 0.19, rho = 0.346). Likewise, parasitism rates were not higher at higher host densities (Spearman's rank test: p = 0.38, rho = −0.192). Bruchid females developing in the field were smaller at high parasitoid population densities (Spearman's rank test females: p = 0.01, rho = −0.503). In contrast, male weight was not significantly reduced at high parasitoid densities (Spearman's rank test: p = 0.08, rho = −0.372).

### Correlations between insect performance and population densities

We found no evidence that bean populations, from which larger beetles or parasitoids emerged, supported higher population densities in the field. No performance parameter of *A. obtectus* measured in the laboratory experiment correlated significantly with *Acanthoscelides* spp. field infestation levels (Table 4). Additionally, the mean weight of *Acanthoscelides* spp. in the field did not correlate with field infestation levels (females: p = 0.98, rho = 0.006; males: p = 0.73, rho = 0.08). Parasitoid sizes measured in the experiments showed no correlation with parasitoid population densities in the field (Spearman's rank test: p = 0.93, rho = 0.02) and bean populations with larger *H. missouriensis* females did not have higher parasitoid population densities (Spearman's rank test: p = 0.33, rho = −0.259).

## Discussion

### Performance of bruchid beetles and parasitoids in the experiment and field

Our results provide evidence that bottom-up (bean population) and top-down factors (presence of parasitoids) both influence the performance of *A. obtectus* at the individual level in the laboratory and in the field. Consistent with our hypotheses, beetle fitness components like weights, developmental time and survival were strongly affected by both the bean population and the presence of the parasitoid *D. basalis*. Our results confirm previous studies showing that those factors can influence herbivore performance simultaneously [3], [5], [53], [54]. Varying nutritional quality of seeds is a likely explanation for the observed variation in *A. obtectus* performance among bean populations. The correlation of bruchid weights across bean populations between laboratory and field is a strong indication that nutritional quality of beans is also important in determining the performance and fitness of bruchids in the field. Other studies have confirmed that differences in nutritional quality (mostly due to allelochemicals) can affect the development of herbivores and also their natural enemies [9], [20], [24], [55]. The domestication status of wild beans has been shown in laboratory experiments to be important in determining performance of herbivores and natural enemies [55]. Bruchid beetles of the genus *Zabrotes* and their parasitoids performed better on cultivated than on wild *Phaseolus* plants presumably because cultivars have lower concentrations of toxic allelochemicals and thus were easier to digest. Wild populations of common beans, *P. vulgaris*, vary in their seed defence protein contents and mainly arcelin has been found to affect bruchid beetle performance [27], [28], [30], [56]. In another study [57] we have analysed the seed protein contents of some of the bean populations used in the performance experiments and found that arcelin is present and might be responsible for the low bruchid beetle performance in the bean population “QUES”. However, we found no indications for the presence of arcelin in any other bean population. Therefore we conclude that there are components in bean seeds, other than arcelin, which are responsible for differences in plant quality among populations. For example, the concentration of phaseolin, which is the main seed storage protein and easily digestible, has been shown to be important for larval development [58], [59]. The seed coat is another bean characteristic which can affect beetle larval performance. As a chemical (lignin content, biting-deterrents) or mechanical barrier (hardness) it might influence the penetration success of first instar larvae and thus affect *A. obtectus* performance and adult emergence rates [60], [61], [62]. Stamopoulos et al. [61] have demonstrated that lignin of *P. vulgaris* teguments, which have been incorporated in artificial diets, can have negative effects on weights of emerging *A. obtectus*.

However, in contrast to other findings, we found no evidence that the influence of plant nutritional quality on the herbivores' performance was passed on to the third trophic level, since we did not find larger parasitoids on larger hosts, neither in the laboratory nor in the field. Likewise, no indications were found for correlations between host and parasitoid developmental times. This is in contrast to other study systems in which qualitatively superior host plants supported larger herbivores and thus larger parasitoids [18], [19], [63], [64] and where the parasitoids developmental time was correlated with its hosts' developmental time [65]. Apparently differences in nutritional quality between bean populations, which affected bruchid beetle performance, did not alter the quality of beetles as hosts for parasitoids. The finding that parasitoid sizes in the experiment did not correlate with those in the field further indicates that parasitoid performance is governed more by other factors than the observed variation in host performance. These results indicate that although plant quality might affect herbivore performance in some cases, this effect is not necessarily passed on to higher trophic levels. However, some of the correlations might have a low statistical power due to the low numbers of parasitoids that emerged from some field samples (Table 1). Variation in plant quality can affect different trophic levels to different extents and the strength of these effects can decrease along the food chain [13], [55], [66]. Velten et al. [30] concluded that arcelin in common bean seeds, which affected *A. obtectus* performance, caused no direct effects on *D. basalis* progeny fitness and size (tibia length, head width) and parasitoids were able to develop on all tested bean lines containing different arcelin concentrations. Also, size might not always be the best indicator of parasitoid fitness. For example, under bean storage conditions, *D. basalis* shows a very good capacity to move through the seed column and therefore to locate hosts [67]; here, being too large may actually be disadvantageous for females. It should also be mentioned that increases in body size need sometimes to be traded-off with other parameters affecting fitness (e.g. suitability of habitat conditions, survival) or may simply be physiologically constrained [68].

In the laboratory, apart from parasitoids directly killing their hosts, we found indirect, non-lethal top-down effects on bruchids, which resulted in reduced beetle weights and shorter developmental times in the presence of parasitoids. These findings were partly confirmed in the field, where female beetles were remaining smaller at high parasitoid densities (indicated by high parasitism rates); for males this trend was also present, but just not significant. Reduced feeding activity of beetle larvae in the presence of natural enemies could explain smaller weights of adult beetles. Vibrations, that are unavoidable during host searching by parasitoids, can be used by insect larvae living in the substrate to detect the presence of their enemies [69], and arresting feeding themselves prevents emitting vibrations that would give away their presence to foraging wasps [70]. Bruchid larvae also started pupation earlier in the presence of parasitoids, which shortens the time they are exposed to parasitoid attack. The benefit of such anti-predatory behaviours is a lower risk of being killed; the cost however is a usually lower energy intake rate, which results in smaller sizes and reduced fecundity [31]. Such indirect trait-mediated predator effects can be as strong as direct lethal effects in influencing herbivore communities [37], [38]. Skelly and Werner [33] have shown that tadpoles metamorphosed at smaller sizes, when predators were present and they have argued that predators are important in structuring the behaviour and life-historical attributes of prey, even without considering lethal effects.

Reduced *A. obtectus* weights in the presence of *D. basalis* could also be explained by oviposition preferences of parasitoids for larger hosts, because in our experiments we didn't prevent parasitoids from laying eggs. Larger hosts are a larger resource for the developing parasitoids and could thus be more profitable than small ones [71], [72] or easier to locate for parasitoid females [73]. However, we find it unlikely that *D. basalis* females in the experiment showed a strong preference for larger hosts because of several reasons. First, we did not find a correlation between host size and parasitoid size, indicating that parasitoids do not actually grow larger on large hosts, and therefore there is no obvious reason to prefer larger hosts. Second, *D. basalis* females anesthetize the host larvae before depositing the eggs thereby preventing further larval growth after parasitism [74]. Thus, if parasitoids would preferentially lay eggs on large hosts and there would be no non-lethal negative effects on bruchid larvae, the fast-developing larvae would be parasitized first and the remaining slow-growing larvae, that escaped parasitism, would develop to their final size, which is reached at a later time. This would actually increase larval developmental times while having no strong effect on final host size. Our results suggest the opposite: reduced developmental times and smaller final size in the presence of parasitoids. Thus, we conclude that we found strong indications for the presence of trait-mediated indirect top-down effects. A decisive experiment would be to use parasitoids which can search for hosts, but are not allowed to oviposit; however, this is technically difficult to reach.

### Correlations between performance of insects and densities in the field

We found no evidence that the better performance of bruchids or parasitoids on certain bean populations would lead to an increase in field abundances. Measured field weights, parasitoid sizes and bruchid experimental performance components (weight, developmental time and emergence rates) showed no correlation with bruchid field infestation levels or parasitism rates. In contrast, other studies have shown that herbivore population sizes or population growth rates are bottom-up regulated and differ between plant genotypes [9], [24], [25]. Aphid colony sizes in field systems were found to be more bottom-up regulated by plant genotype, while natural enemy abundance was unaffected by the plant secondary metabolites [9]. Johnson [25] found evidence that plant genotype had a significant direct effect on the abundance of natural enemies, irrespective of the herbivore density and an indirect effect, mediated through herbivore density. However in our study, parasitism rates did not depend on host densities (bruchid infestation levels). This result is consistent with other study systems in which the percentage of parasitized hosts was density-independent [23].

It appears that other factors, for example environmental stochasticity or the surrounding landscape, have a more important effect on population densities in this system than top-down or bottom-up effects. It has been shown that Mexican bean weevil, *Z. subfasciatus*, females show behavioural and physiological plasticity in oviposition behaviour according to host availability [75]. When beans were scarce and competition was high, beetles laid more eggs onto the same seed and fewer, less fecund adults emerged. This could have strong impacts on beetle population densities when plant resources vary in their availability from season to season, as it seems to be the case in wild beans (personal observation). Bowler and Benton [76] have shown that soil mite populations, which experienced variation in daily food supply (variable food availability versus constant food supply), had lower and more variable population densities than populations in a constant environment. Environmental variation has been shown to be important in influencing population dynamics [77]–[79]. Mutshinda et al. [79] have found, when analysing community time series among different taxa that population dynamics were dominated by environmental stochasticity. This accounted for 40–95% of the temporal variances in individual species abundances. However, the variability of an environment, expressed as for example in resource stochasticity, is an important factor in determining insect population abundances [80]. One limitation of our study is that we collected field data from only one year. In order to measure the effect of environmental stochasticity on population dynamics over time, it would be necessary to analyse insect abundance data from several years.

Parasitoids of the genus *Horismenus* are important natural enemies of bruchid beetles, but little is known about their biology and most species of the Neotropical region remain undescribed [43], [81]. *Horismenus missouriensis* is a generalist parasitoid of Coleoptera, Lepidoptera and Diptera [82]. Since those parasitoids are generalists and accept a wide range of hosts, they do not depend upon the density of one particular host species. This could be another explanation why parasitoids were unaffected in their densities of *Acanthoscelides* spp. field infestation levels.

## Conclusions

Our study shows that plant quality (bottom-up) and natural enemies (top-down) act together in influencing performance of a herbivore, confirming previous studies. We also showed that enemies can have direct and indirect, non-lethal effects on individual host performance. However, the bottom-up effects of plant quality on individual bruchid beetles was not passed on to the third-trophic level. Up to now, few laboratory experiments and field studies have investigated and provided evidence for plant quality effects cascading up to higher trophic levels [13], [25], [64], [65], [83]. Although in some systems, plants might affect natural enemies; apparently in our study, factors other than plant quality are more important in determining parasitoid performance. Furthermore, we found that increased individual performance does not necessarily translate into increased densities at population levels. Our results are in contrast to other studies, which provided evidence for bottom-up regulation of herbivore populations by plant genotype [25]. A plausible explanation is that in our system environmental variation can have a larger impact on insect communities than biotic interactions. We conclude therefore that it is important to differentiate between effects acting on individual insects and those acting on insect population levels. Factors influencing lower trophic levels do no necessarily cascade-up to higher trophic levels and increased individual fitness does not necessarily result in increased population densities. However, to elucidate the effect of environmental stochasticity on abundances of bruchid beetles and their natural enemies, long-term studies are needed.
